# Influenza-Associated Benign Acute Childhood Myositis During the 2024–2025 Season: A Retrospective Multicenter Study

**DOI:** 10.3390/children12101333

**Published:** 2025-10-04

**Authors:** Chrysoula Kosmeri, Margarita Efthalia Papasavva, Afroditi Kyrkou, Vasiliki Gketsi, Ekaterini Siomou, Fani Ladomenou, Alexandros Makis

**Affiliations:** 1 Department of Pediatrics, University Hospital of Ioannina, 45500 Ioannina, Greece; 2 Department of Pediatrics, G. Hatzikosta Hospital, 45445 Ioannina, Greece; 3 Faculty of Medicine, School of Health Sciences, University of Ioannina, 45500 Ioannina, Greece

**Keywords:** influenza, benign acute childhood myositis, pediatric complications, creatine kinase, viral myositis, influenza B

## Abstract

**Highlights:**

**What are the main findings?**

- BACM occurred in one-third of hospitalized children with influenza, mainly inschool-aged children.
- There was a strong association with influenza B.

**What is the implication of the main finding?**

- Despite dramatic CK elevations, BACM has a benign course with supportive management, suggesting that awareness of its characteristic presentation may help avoid unnecessary interventions and reassure families.
- Vaccination, especially quadrivalent, may reduce BACM incidence.

**Abstract:**

**Background**: The aim of this study was to describe the clinical and laboratory characteristics of hospitalized pediatric influenza cases during the 2024–2025 season in Northwestern Greece, with a focus on influenza-associated benign acute childhood myositis (BACM). **Methods**: We conducted a retrospective observational study of children aged 0–16 years hospitalized with laboratory-confirmed influenza between October 2024 and May 2025 at two pediatric departments. BACM was diagnosed based on calf pain, difficulty walking, elevated creatine kinase (CK), and symptom resolution without other causes. **Results**: A total of 113 children (mean age 7.0 ± 4.2 years; 50.4% male) were included; 61.1% had influenza A and 38.9% influenza B. None had received influenza vaccination. BACM was identified in 37 children (32.7%), who were significantly older than patients without myositis (9.3 ± 2.7 vs. 6.0 ± 4.5 years, *p* < 0.001). Influenza B was strongly associated with BACM (70.3% vs. 29.7%, χ^2^(1) = 22.7, *p* < 0.001, Cramer’s V = 0.448). Median CK in BACM cases was 2637 IU/L (range: 189–129,390 IU/L); all had preserved renal function. One patient with congenital myopathy developed rhabdomyolysis (peak CK 130,000 IU/L) but had a full recovery. All patients received oseltamivir and supportive care; no intensive care admissions or deaths occurred. **Conclusions**: In our hospitalized cohort, BACM was observed relatively frequently (32.7%), particularly in children with influenza B; however, this proportion reflects hospitalized cases and does not indicate the true incidence in the general pediatric population. Despite high CK levels, outcomes were favorable with supportive care. These findings underscore the importance of clinician awareness to avoid unnecessary investigations and hospitalizations.

## 1. Introduction

Influenza is a common viral infection in children, primarily caused by influenza A and B viruses. Children play a significant role in the transmission of influenza within communities, acting as key vectors of the disease. Transmission occurs through respiratory secretions, contaminated surfaces, and aerosolized particles expelled during breathing [1,2]. Influenza epidemics occur annually, especially in temperate regions during the winter months, although the specific timing and duration of the influenza season vary by country and year.

Influenza in children can present with a wide spectrum of clinical manifestations, ranging from mild upper respiratory tract symptoms to more serious conditions such as bronchiolitis, pneumonia, and severe complications [3]. While the illness is typically self-limiting in otherwise healthy children, it can cause substantial morbidity and mortality, particularly in high-risk populations [4].

One rare but serious complication is benign acute childhood myositis (BACM). The diagnosis of this condition is based on the recent influenza infection, clinical findings of sudden onset of bilateral calf pain, impaired gait, or refusal to walk, and confirmatory laboratory findings of elevated serum creatine kinase (CK) levels [5,6]. BACM usually emerges during the convalescent phase of the febrile illness and is clinically distinct from the generalized myalgia commonly associated with early influenza symptoms [5,6,7].

Although the true incidence of influenza-associated BACM is not well defined, a five-year retrospective study found that influenza accounts for approximately 80% of all viral BACM cases annually [8]. In another retrospective analysis conducted at the Department of Pediatrics, Wonkwang University Hospital (2010–2016), 47 out of 536 children diagnosed with influenza B infection reported bilateral calf pain with or without difficulty walking [9].

Due to its infrequent occurrence and overlap with more serious neurologic or autoimmune conditions, such as cerebellar ataxia, cerebellitis, or Guillain–Barré syndrome, influenza-associated BACM may be under-recognized by clinicians. This misrecognition can lead to unnecessary hospital admissions and extensive diagnostic investigations [8,10].

The objective of this study is to document and analyze cases of influenza during the 2024–2025 winter season in two hospitals in Northwestern Greece. Particular emphasis is placed on evaluating the clinical and laboratory characteristics of influenza-associated BACM in the pediatric population.

## 2. Materials and Methods

### 2.1. Study Design and Population

This is a retrospective observational study conducted in the Department of Pediatrics at two reference hospitals in Northwestern Greece. The study included all children hospitalized with laboratory-confirmed influenza infection during the winter period of October 2024 to May 2025. Data were collected through a review of electronic medical records and discharge summaries.

Children 0–16 years with a confirmed diagnosis of influenza who were admitted to either of the two hospitals were eligible for inclusion. A total of 113 pediatric patients were included. Influenza diagnosis was established using either rapid antigen testing (Dr. Care Antigen Test Kit by Emoria Healthcare) or a commercial multiplex RT-qPCR (reverse transcription quantitative real-time Polymerase Chain Reaction) assay on nasopharyngeal swab specimens depending on clinical availability and physician decision.

### 2.2. Data Collection

Demographic, clinical and laboratory data were extracted from medical records using a standardized data collection form. A complete patient history was recorded including demographic data, history of underlying diseases, history of vaccination against influenza, and history of prior severe infections or coinfections during hospitalization, as well as hospitalization duration.

In each case clinical manifestations were noted, including common and rare symptomatology but also manifestation of complications. Regarding laboratory findings, blood differential count, inflammatory markers, creatinine, CK, lactate dehydrogenase (LDH), aspartate aminotransferase (AST) and alanine aminotransferase (ALT) were noted. Moreover, the type of influenza, as influenza A or influenza B, as well as the serotype of influenza A when available, were recorded. Further diagnostic evaluations included findings from chest X-rays and electrocardiograms, which provided insight into respiratory and cardiac involvement. Information regarding duration of hospitalization, administration of oseltamivir, other treatment therapies, and outcome were also noted.

For a subgroup analysis, cases of BACM were identified based on the combination of calf pain, difficulty walking, elevated CK, and clinical resolution within days without other identifiable causes. Neither electromyography nor MRI was performed, as these investigations were not routinely required for typical cases of BACM. Additionally, demographic data, hospitalization duration, clinical and laboratory findings, CK levels, renal function, complications, and treatment outcomes were also collected.

### 2.3. Statistical Analysis

Descriptive statistics were used to summarize patient characteristics. Continuous variables were expressed as mean ± standard deviation (SD) or median with interquartile range (IQR), as appropriate. Categorical variables were presented as frequencies and percentages. Chi-square tests were used to assess associations between categorical variables, such as influenza type and the presence of BACM. For comparisons of continuous variables (e.g., age and hospitalization duration), independent samples t-tests were used. A *p*-value of <0.05 was considered statistically significant. Effect size for significant associations was assessed using Cramer’s V. Statistical analyses were conducted using SPSS software (version 29.0; SPSS Inc., Chicago, IL, USA).

## 3. Results

A total of 113 pediatric patients with laboratory-confirmed influenza infection who were hospitalized at the two participating centers were included in the study. The mean age was 7.0 ± 4.2 years (range: 1 month to 16 years), with a near-equal sex distribution (50.4% male). A minority of children (14.2%) had known underlying medical condition, specifically congenital myopathy, Wiedemann–Steiner syndrome, asthma, congenital heart disease, congenital anomalies of the kidney and urinary tract (CAKUT) and prematurity.

The mean duration of hospitalization was 4.0 ± 1.7 days. Notably, none of the children had received seasonal influenza vaccination prior to their illness. Regarding virological findings, 61.1% were diagnosed with influenza type A, and 38.9% with type B. Only 13.3% of cases were diagnosed using PCR testing; the remainder were diagnosed via rapid antigen tests, limiting subtype identification for influenza A. Almost half of patients (46.4%) had documented exposure to a confirmed influenza case. Previous respiratory hospitalizations were rare (5.5%), and coinfections during the current illness were observed in 5.6%. Specifically, three bacterial coinfections (including Streptococcus spp., Chlamydophila pneumoniae, and possible bacterial pneumonia) and three viral coinfections (including RSV, parainfluenza, and) were identified. Fever was the most common symptom, followed by cough and gastrointestinal symptoms (abdominal pain, vomiting, diarrhea). The most frequently observed complication was BACM (32.7%), followed by pneumonia (16.8%).

All patients received oseltamivir during hospitalization and recovered fully without complications. Of note, 23% had initiated oseltamivir prior to admission, prescribed by their primary care pediatricians.

### Benign Acute Childhood Myositis (BACM)

A total of 37 children (32.7%) were diagnosed with acute BACM. The mean age was 9.3 ± 2.6 years and 56.8% were male. The youngest case involved a 5-year-old child. The mean duration of hospitalization in this group was 4.08 ± 2.18 days. All children with BACM presented during the febrile stage of influenza infection, with calf pain and walking difficulties coinciding with fever at admission. Demographics, clinical characteristics, laboratory findings and complications of children with BACM compared to those without BACM are presented in Table 1. Among children with BACM, four had underlying conditions: congenital myopathy, urogenital malformation, allergy, and autism.

Influenza B was significantly more common among children with BACM (70.3%) than type A (29.7%). Statistical analysis revealed a significant association between influenza type and development of BACM (χ^2^(1) = 22.7, *p* < 0.001, Cramer’s V = 0.448), indicating a higher likelihood of BACM among children with type B influenza.

Children with BACM were significantly older than non-BACM children. No statistically significant difference was found in the duration of hospitalization in children with BACM compared to those without. There were no significant differences in sex distribution or presence of underlying conditions between children with and without. None of the children diagnosed with BACM had a concurrent coinfection.

The clinical presentation of BACM cases was remarkably uniform. All patients experienced bilateral calf pain and difficulty walking, accompanied by fever at admission. Additional symptoms included cough and gastrointestinal manifestations such as abdominal pain, diarrhea, and vomiting.

CK levels were markedly elevated in children with BACM, with a mean value of 8395 ± 21,405 IU/L, a median of 2637 IU/L, and a wide range (189 to 129,390 IU/L; IQR: 3303). All children had normal creatinine levels and no cases of renal impairment were observed. Two children had a prior history of similar post-viral BACM episodes, with negative workups for underlying myopathies.

A 7-year-old boy with underlying congenital myopathy and a history of recurrent viral-associated BACM presented during the current influenza illness with clinician-diagnosed rhabdomyolysis. Symptomatology included generalized muscle pain affecting the calves, thighs, upper arms and abdominal muscles (tender to palpation). Laboratory evaluation revealed a peak CK of 130,000 IU/L and myoglobinuria, but he developed neither renal complications nor compartment syndrome and had a favorable outcome with complete recovery.

Additional complications in the BACM group included one case of pneumonia and one case of myocarditis. All children received oseltamivir, and one-third (33.3%) had initiated treatment prior to admission. Management was supportive, mainly involving intravenous hydration. All patients experienced full clinical recovery with supportive care, without the need for intensive care.

## 4. Discussion

This study offers a comprehensive overview of the clinical and laboratory characteristics of hospitalized children with influenza-associated BACM. Consistent with prior reports, BACM was strongly associated with influenza B, with up to 86% of cases in the literature linked to this type. In our cohort, BACM was observed predominantly in school-aged children (mean age 9.3 years), with no cases identified under 5 years of age. This age distribution aligns with prior reports suggesting that BACM is most frequent in older children and rarely occurs in younger ones, possibly reflecting developmental differences in muscle susceptibility or the ability to verbalize symptoms [8,11,12,13]. BACM is rare in adults and this is a noteworthy observation that may be due to a high physical activity in children with a reluctance to rest. Although earlier studies described a male predominance [5,8,14], we did not observe significant sex differences between BACM and non-BACM patients. Due to the small number of coinfections overall, our data did not allow for conclusions on whether coinfections could influence the development of BACM.

Clinical presentation was also consistent with the literature. The hallmark symptoms of bilateral calf pain, difficulty walking, and preceding febrile illness were frequently observed [12,15]. Laboratory findings further reinforced the diagnostic profile of BACM since CK elevations were nearly universal, as reported in previous studies [5,12,15]. Elevated CK concentrations are a valuable diagnostic marker, with declining levels typically correlating with clinical recovery. In our cohort, blood CK levels were markedly elevated, demonstrating a wide range and reaching values as high as 129,390 IU/L. Importantly, despite dramatic biochemical abnormalities, all cases achieved full clinical recovery, typically within a week, as described in earlier work [16,17].

The exact pathogenesis of BACM remains undetermined. Several theories have been proposed, including immune-mediated muscle inflammation and direct viral invasion [18]. Evidence of viral proteins in muscle tissue and successful infection of human myocytes in vitro supports the possibility of direct myocyte infection [19]. However, limited pathological findings of inflammation in muscle biopsies suggest an immune-mediated process may also contribute [19,20].

Management and outcomes in our cohort reaffirm the benign nature of BACM. Supportive care including intravenous hydration, pain control, and monitoring of urine output was sufficient, while no child needed intensive care or prolonged hospitalization. Although oseltamivir was administered in all hospitalized children and to one-third prior to admission, existing evidence suggests only modest reductions in influenza symptom duration [12].

Prevention through vaccination remains a critical challenge. As stated above no children in our cohort were vaccinated against influenza, which is consistent with broader observations indicating low influenza vaccination coverage in pediatric populations, particularly in those diagnosed with influenza [21,22]. The 2024–2025 National Immunization Program recommends influenza vaccination for all healthy children aged 6 months–5 years and for older children in high-risk groups. Given the established association between influenza B and BACM, the use of quadrivalent influenza vaccines, which protect against both B lineages, may be important in reducing BACM incidence, though further research is needed to confirm this [8].

Recurrence and complications were rare but noteworthy. Two children had previous episodes of viral-associated BACM, with negative investigations for underlying myopathies, while a child with a history of congenital myopathy had several prior episodes of viral-associated BACM. In the literature, up to 9.8% of children have recurrent episodes, usually in otherwise healthy patients, with a clinical course similar to primary cases [13]. Complications from BACM are exceptionally rare. Although rhabdomyolysis and acute kidney injury have been reported, their occurrence is low [5,8,23]. In one cohort of 52 influenza-associated BACM, only a single case of rhabdomyolysis (<0.5%) occurred without renal involvement [8]. A review of 316 cases found rhabdomyolysis in 3% (predominantly girls, mostly influenza A), with renal failure in 80% of those cases [5]. However, this analysis was based on aggregated case reports, and the elevated proportion of severe outcomes may reflect publication bias toward more atypical or critical presentations. Other systematic reviews and retrospective studies similarly confirm that progression to renal failure is exceedingly rare, with many large cohorts reporting no cases at all [11,12,13].

Although systemic muscle effects can occur with various febrile viral infections, BACM is a distinctive complication of influenza. The novelty of our study lies in documenting its frequency and characteristics within a hospitalized pediatric cohort during a single influenza season, thereby providing real-world data to complement previous reports.

Limitations and future directions should be acknowledged. First, our findings do not capture the true incidence of BACM, since hospitalized cohorts are biased toward more severe cases and admission practices vary by region. Hospitalization rates for BACM vary widely across studies and may reflect differences in local admission practices, study designs, and clinician familiarity with the condition. Intense pain and difficulty walking can prompt admission for observation and supportive care, even in otherwise stable patients. The youngest identified BACM case was 5 years old. However, it is worth noticing that the diagnosis in infants and very young children is challenging, as they may not be able to verbalize pain or demonstrate typical walking difficulties, therefore it is possible that mild cases of BACM in this age group occurred but did not require hospitalization and therefore were not captured in our cohort. Second, the retrospective design limited our ability to standardize data collection, particularly regarding viral subtyping and outpatient cases. Finally, the relatively small sample size restricts subgroup analysis, for example, on sex differences or recurrence risk. Future research should focus on multicenter prospective studies to better define incidence, identify risk factors for recurrence, and clarify the role of vaccination in BACM prevention. Studies exploring viral–muscle interactions could also provide insightful information on pathogenesis.

## 5. Conclusions

In conclusion, our findings reaffirm BACM as a recognizable, self-limiting complication of influenza, particularly influenza B, in school-aged children. Despite high CK levels and dramatic clinical presentations, the prognosis is excellent with supportive care. Improved vaccination coverage—especially with quadrivalent formulations—holds promise in reducing both the incidence of influenza and its musculoskeletal complications like BACM.

## Figures and Tables

**Table 1:** Main characteristics of hospitalized children with influenza-associated BACM compared to those without BACM.

	Children with BACM (*n* = 37)	Children Without BACM (*n* = 76)	*p*-Value
**Main feature**	**n (%) or Mean ± SD**	**n (%) or Mean ± SD**	
Male sex	21 (56.8%)	36 (47.4%)	0.46
Age (years)	9.3 ± 2.6	6.0 ± 4.5	<0.001
Underlying condition	4 (10.8%)	12 (15.8%)	0.67
Duration of hospitalization (days)	4.08 ± 2.18	4.2 ± 1.5	0.7
Influenza B	26 (70.3%)	18 (23.7%)	<0.001
Influenza A	11 (29.7%)	58 (76.3%)	<0.001
Coinfections	0	6 (8.1%)	0.17
**Clinical symptoms**	**n (%)**	**n (%)**	
Fever	37 (100%)	74 (98.7%)	1
Cough	24 (35.1%)	50 (66.7%)	1
Respiratory distress	0	6 (8%)	0.18
Gastrointestinal symptoms	7 (18.9%)	32 (42.1%)	0.026
Chest pain	1 (2.7%)	4 (5.3%)	0.9
**Laboratory findings**	**n (%) or Mean**	**n (%) or Mean**	
Leukopenia	16 (43.2%)	8 (10.7%)	<0.001
Neutropenia	21 (56.8%)	16 (21.3%)	<0.001
Lymphopenia	15 (40.5%)	24 (32%)	0.49
Increased CRP levels	1 (2.7%)	11 (14.5%)	0.1
Increased hs-TPN levels	3 (2.7%)	0	0.033
CK levels (IU/L)	8395 ± 21,405	130 ± 96	<0.001
Creatinine levels (mg/dL)	0.69 ± 0.12	0.6 ± 0.17	0.065
**Complications**	**n (%)**	**n (%)**	
Pneumonia	1 (2.7%)	18 (23.7%)	0.011
Myocarditis	3 (2.%)	0	0.033

BACM: benign acute childhood myositis, CRP: C-reactive protein, CK: creatine kinase, hs-TPN: high sensitivity troponin.

## Data Availability

The original contributions presented in this study are included in the article. Further inquiries can be directed to the corresponding author.

## Abbreviations

The following abbreviations are used in this manuscript:

BACM	benign acute childhood myositis
CK	creatine kinase
hs-TPN	high sensitivity troponin
IU/L	International Units per Liter
PCR	polymerase chain reaction
